# The Vending and à la Carte Policy Intervention in Maine Public High Schools

**Published:** 2005-10-15

**Authors:** Anne-Marie Davee, Janet E Whatley Blum, Rachel L Devore, Christina M Beaudoin, Lori A Kaley, Janet L Leiter, Debra A Wigand

**Affiliations:** Muskie School of Public Service, University of Southern Maine; Department of Sports Medicine, University of Southern Maine, Gorham, Me; Department of Sports Medicine, University of Southern Maine, Gorham, Me; Department of Sports Medicine, University of Southern Maine, Gorham, Me; Muskie School of Public Service, University of Southern Maine, Augusta, Me; Maternal and Child Health Nutrition Program, Bureau of Health, Maine Department of Health and Human Services, Augusta, Me; Cardiovascular Health Program, Bureau of Health, Maine Department of Health and Human Services, Augusta, Me

## Abstract

**Background:**

A healthy school nutrition environment may be important for decreasing childhood overweight. This article describes a project to make healthier snacks and beverages available in vending machines and à la carte programs in Maine public high schools.

**Context:**

Seven public high schools in Maine volunteered to participate in this project. Four schools made changes to the nutrition environment, and three schools that served as controls did not. The nutrition guidelines were to offer only low-fat (not more than 30% of total calories from fat) and low-sugar (not more than 35% by weight of sugar) items in vending machines and à la carte programs.

**Methods:**

Strategies to implement the project included early communications with school officials, monetary stipends for participation, identification of a school liaison, and a committee at each school to promote the healthy changes. Baseline nutrient content and sales of all competitive foods and beverages were assessed to develop the guidelines for changes in the four schools. Student volunteers at all seven schools were measured for height, weight, diet quality, and physical activity level to assess the impact of the change to the nutrition environment. Baseline measures were taken in the spring semester of 2004. Nutrition changes were made to the à la carte programs and vending machines in the four intervention schools at the start of the fall semester of 2004. Follow-up nutrition assessment and student data collection occurred in the spring semester of 2005.

**Consequences:**

Healthy changes in vending machines were more easily achieved than those made in the à la carte programs. Technical assistance and ongoing support were essential for successful implementation of this intervention.

**Interpretation:**

It is possible to improve the nutrition environment of Maine public high schools. Stakeholder support is essential to sustain healthy changes.

## Background

The statistics on youth overweight, levels of physical inactivity, and poor food choices have become a public health concern. The prevalence of overweight among U.S. children has more than doubled in the last 20 years and tripled among adolescents (1). Overweight in youth is related to numerous long-term health consequences, including elevated blood cholesterol levels and high blood pressure, and may precipitate immediate ailments such as respiratory disorders, orthopedic conditions, and hyperinsulinemia (2).

Schools play an important role in influencing the diet of children and adolescents. Schools reach more than 95% of youth between the ages of 5 and 17 years (2). More than 50% of youth in the United States consume one of three meals in school, and 10% eat two of three meals in school (2). Students consume 25% to 33% of their daily calories at school (3). Therefore, efforts to monitor and improve the nutritional quality of food choices in schools are indicated (3). A healthy school nutrition environment helps students adopt and maintain healthy eating behaviors and promotes academic achievement (4).  

Because of the upward trend in childhood overweight, concern has arisen about the nutritional quality of foods and beverages sold in schools outside of federally regulated meal programs. Reimbursable school meals offered through the U.S. Department of Agriculture's National School Lunch Program (NSLP) must meet federally mandated nutrition guidelines, whereas foods and beverages sold outside of the NSLP — known as *competitive* foods — are not required to do so (5). Nationally, 51% of school-aged children eat less than one serving of fruit per day, and 29% eat less than one serving a day of vegetables that is not fried (4). Depending upon age and sex, 56% to 85% of children consume soda on any given day (4). Of particular concern is the shift in beverage consumption from milk products to soda and fruit drinks in all age groups (4). It has been reported that vending machines account for an estimated 23% of the foods sold at school (6). Therefore, strategies to moderate fat and added sugar in children's diets should include making changes to food and beverage choices in school vending machines and à la carte programs. Moderating intake of fat and added sugar to achieve caloric balance is consistent with the 2005 *Dietary Guidelines for Americans* (7).

Schools can play a key role in reversing the trends in childhood overweight by establishing school nutrition policies that provide healthy food choices, requiring nutrition education curricula, and encouraging participation in the NSLP (2). Research suggests that environmental approaches in combination with educational approaches may be most effective in bringing about positive changes in students' eating behaviors (8). This article describes an environmental intervention to make healthier snacks and beverages available in vending machines and à la carte programs in Maine public high schools.

## Context

The rates of overweight and at risk for overweight for children and adolescents in Maine correspond with national trends. Thirteen percent of high school students in Maine are overweight, and 15% are at risk for overweight; 13% of middle school students are also overweight, with 18% at risk for overweight (9). Seventy-seven percent of high school students in Maine do not eat the recommended five servings of fruit and vegetables each day (9). To promote better nutrition at school, Maine requires that competitive foods sold during the school day meet a 5% minimal nutritional value rule; this rule means that each vending machine or à la carte item must provide at least 5% of the Reference Daily Intake (RDI) for one of eight specified nutrients per 100 calories. Despite this rule, concern remains that students' food and beverage choices are too high in fat, sugar, and calories.

To address this concern, a project was developed through the Maine Bureau of Health's Physical Activity and Nutrition Program to make nutrition changes in vending and à la carte programs in Maine public high schools and to assess the impact of the changes on student diet and health. This project, the Vending and à la carte Policy Intervention, was funded through the Centers for Disease Control and Prevention's (CDC's) Obesity Prevention Program. The nutrition guidelines stated that only low-fat items (no more than 30% of total calories from fat, excluding fat from nuts, seeds, and peanut butter) and low-sugar items (no more than 35% by weight of sugar, excluding sugars found naturally in fruit and dairy products) could be sold in high school vending machines and à la carte programs. The guidelines were developed following a review of recommendations from the Maine Dietetic Association and Maine School Food Service Association (10) and other sources; the guidelines are consistent with the CDC's sample list of vending machine food and beverage choices that are low in saturated fat (2). Guidelines for maximum portion sizes in each category of items were also developed. No changes were made to the NSLP. 

In November 2003, the Maine Department of Education sent out an informational letter electronically to all public school districts in the state asking for voluntary participation either to implement nutritional changes or make no changes for one school year in their vending and à la carte programs. Interested high schools were screened to meet project criteria, which included participation in the NSLP and the presence of at least one student beverage and one student snack vending machine. Seven public high schools volunteered to participate and met the criteria. These schools were located in six counties throughout the southern and central regions of Maine. Community populations in these locations ranged from 2500 to 23,000 (11). According to the U.S. Census Bureau's Census 2000, median household incomes for these communities ranged from $28,390 to $56,491 (12).

Four of the seven schools volunteered to implement the nutrition recommendations in vending and à la carte programs, while three schools agreed to serve as controls and make no changes for one school year. All seven schools had a similar variety of foods and beverages available in vending and à la carte programs, with an average of 10 vending machines at each site. Among the schools, approximately 46% of the beverage items and 20% of the snack items sold in vending machines met the program nutrition guidelines, while an estimated 31% of à la carte items met the guidelines. Average high school student enrollment was 800, with an average of four (25-minute) lunch periods and an average of 458 students per day eating in each school cafeteria.

## Methods

This nutrition project had a prospective, quasi-experimental nonrandomized design. It was approved by the Institutional Review Board (IRB) of the University of Southern Maine and by the Bureau of Health, Maine Department of Health and Human Services. The project team consisted of four individuals: an intervention coordinator, a registered dietitian, a research assistant, and a trained project specialist. Strategies to implement the project included early communication between the project team and schools, monetary stipends to participating schools, identification of a liaison at each school, and the creation of a committee at each school to promote the changes.

### School communications

Early in 2004, visits were made to all seven schools to begin communication, obtain the cooperation of school administration, and meet food service personnel. Initial communication focused on introducing the project team, clarifying the objectives of the project, and defining the expectations for participation in the project. Each superintendent signed a contract that required identification of a school liaison, willingness to allow recruitment and measurement of student volunteers, and cooperation with the data collection process.

The liaison identified at each school was responsible for establishing a committee to promote the healthy changes in the vending machines and à la carte menus at their schools. Expectations for the committees were discussed in detail with each school liaison; these expectations included recruitment of representatives from all stakeholder groups — school administration, faculty, students, parents, and food service personnel — and the completion of at least four activities over the course of the school year to promote the healthy changes. A list of recommended activities with resources was provided, and a member of the project team was assigned to attend each committee meeting. All schools received a stipend of $1500 for each school year of participation, a third of which was used to support the liaison.

### Nutrition assessment and implementation of changes

Before implementation of any nutritional changes, baseline assessment of the nutritional value of each vending and à la carte food or beverage item was conducted, and sales trends for the à la carte programs were documented. The data collection occurred during a 1-week period at each school during the winter/spring semester of 2004. During this period, the project team communicated regularly with the school liaison, faculty, food service staff, and students eating in the cafeterias. In each school's cafeteria, a daily total student lunch count and bag lunch count was also recorded. 

Student volunteers were recruited through presentations in selected classes as well as through schoolwide posters and announcements on the public address system. Only freshmen, sophomores, and juniors were eligible because of the 2-year participation requirement. Students were offered a $10 gift certificate to a sporting goods store for each year of participation. Parental and student written consent was obtained before taking measurements. The written consent form included a summary explaining the purpose and plans for the project. A total of 581 students volunteered to participate from the seven schools, an average of 83 students per school; 309 of those students were from the four intervention schools, and 272 were from the three control schools. Student measurements were taken confidentially in the spring semester of 2004.

During the summer of 2004, the project team visited the four schools implementing nutrition changes. These visits included contacts with school administration and the school liaison and meetings with food service staff. The project team also met directly with each food and beverage supplier for each school to present the nutrition guidelines and identify those items that met the criteria. The suppliers were expected to make the changes to vending machine items by the start of the fall semester. Food service managers were given lists of the foods and beverages from their existing suppliers that met the nutrition guidelines. The project team also offered suggestions and recipes for acceptable substitutions for those that did not meet the nutrition criteria.

Presentations that emphasized the potential for the environmental change to have a positive impact on student health were provided for faculty at the start of the school year in each school making changes. In addition, letters were sent home to inform students and their parents of upcoming changes in the vending and à la carte programs. Implementation of the majority of the changes in the vending and à la carte programs occurred on the first day of the 2004–2005 school year so that when students and faculty arrived, primarily low-fat and low-sugar items were available in the vending machines and à la carte programs.

Biweekly then monthly visits were made by a project team member to each school that implemented the changes to provide ongoing technical support throughout the first 6 months of the school year. To maintain communication, visits were also made periodically to the schools that did not make changes. In the spring semester of 2005, a follow-up nutrition assessment of vending machine and à la carte food items was conducted in all seven schools, and data on sales of the à la carte items were collected. Because of the proprietary nature of vending machine sales, the purchase of foods and beverages located in locked vending machines could not be assessed accurately.

## Consequences

Baseline assessments of the vending machines in all seven schools showed that approximately 80% of the snack items and 54% of the beverage items did not meet the nutrition guidelines. Baseline assessment of the à la carte programs showed that an estimated 69% of items did not meet the nutrition guidelines. In the four intervention schools, items in vending machines that did not meet the guidelines were removed and replaced by the suppliers with alternatives that did meet the nutrition criteria, such as granola bars, nuts, dried fruits, pretzels, crackers, and baked chips (Figure). No changes were made to the competitive food choices at the other three schools.

**Figure:**
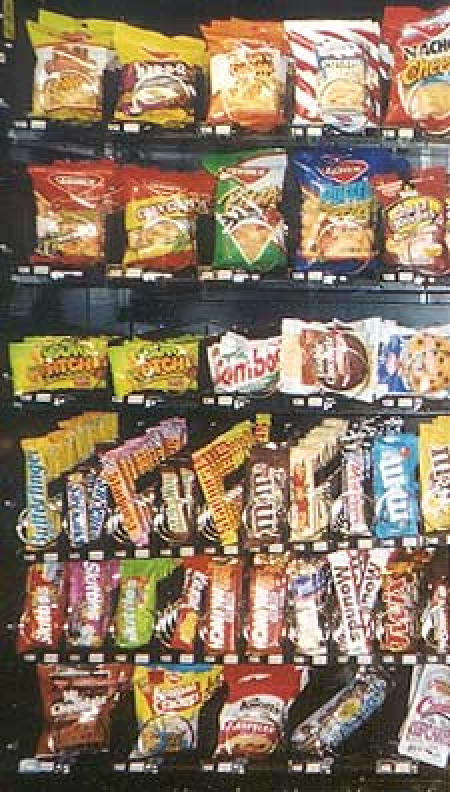

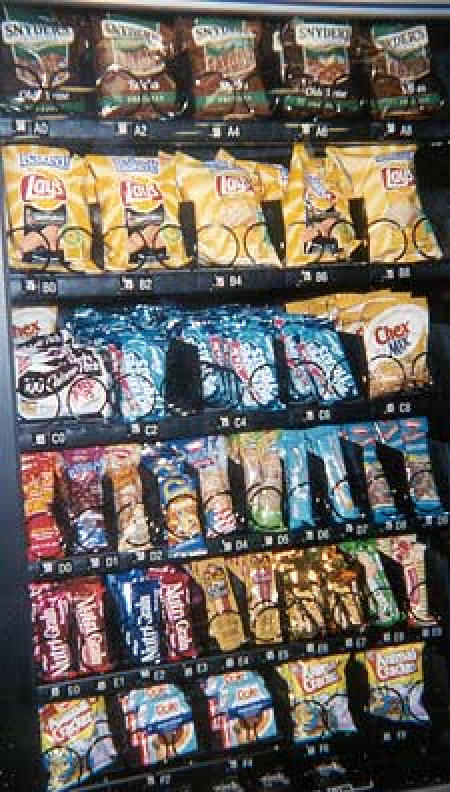
Sample vending machine at a Maine public high school before (left) and after (right) implementation of the Vending and à la carte Policy Intervention, 2004. Only food items that met established nutrition criteria were available in vending machines postintervention.

There were unanticipated barriers to obtaining both the sales and nutritional information for vending machine products. Suppliers did not collect sales information by item nor did they keep inventories of the stock in each machine. Sales data are considered proprietary and were therefore not obtainable from the many vendors in each school. Nutrition facts were not available on some product labels; in those cases, the information was obtained from the suppliers.

### Response to nutrition changes

More than 50 Maine school districts contacted the intervention coordinator to inquire about this intervention after learning about it from the informational letter. This response demonstrated statewide interest in offering healthier food and beverage choices in schools. Because of proposed legislation to improve vending machine options in Maine, the à la carte programs were added to the project design. Adding all à la carte foods and beverages to the project increased the scope of changes required for implementation.

Two schools that implemented the healthy changes in the vending and à la carte programs did so with minimal reactions. In these schools, the school administrators and liaisons actively promoted and supported the change to the nutrition environment with faculty, food service staff, and students with an emphasis on the overall potential for positive impact on student health.

In the other two schools, anecdotal evidence showed that the responses to the change in the nutrition environment by faculty, staff, and students were mixed. Students and faculty reacted most to the nutrition changes in the à la carte programs. Comments were made about the removal of specific items (carbonated beverages, cookies, and high-fat snacks), the perceived lack of food and beverage choices, and smaller portion sizes with similar costs. It was evident that the students and faculty did not anticipate the depth and impact of the changes on their daily food and beverage choices in the school nutrition environment. The changes to vending machine choices, however, did not evoke adverse reactions, with the exception of the elimination of carbonated beverages in the faculty rooms.

In one school, negative responses from students and faculty appeared to have a significant impact on the food service staff. In this school, the food service staff expressed concerns about how the changes made in the à la carte program would affect their daily responsibilities and potentially their job security.

### Committee activities to promote nutrition changes

Committee activities at the four schools that made changes included taste-testing of healthier foods, display of banners encouraging consumption of fruits and vegetables, and visual demonstrations of the amounts of fat and sugar in foods. These activities varied among the four schools by meeting frequency, number of participating stakeholders, and the commitment to the promotion and support of the project. The liaisons at two of the schools changed, and the food service director resigned at one of the schools during the intervention. These circumstances as well as other variables affected the ability of the committees to complete their required activities. A model vending and à la carte nutrition policy was developed for adoption by each school after implementation of the intervention to sustain the environmental change.

## Interpretation

Changes in the nutrition environment to meet guidelines for healthier foods and beverages and smaller portion sizes in à la carte programs and vending machines are achievable in Maine public high schools. Food and beverage suppliers were able to provide items that met the nutrition guidelines. Frequent school visits that included regular communication and ongoing technical assistance were essential during the implementation phase. 

Vending machine changes were easily implemented and well accepted by students. Changes in the à la carte programs were more difficult, especially in the schools lacking support from all stakeholders. Future interventions to change school nutrition environments should consider early communication with all stakeholders, including students, parents, faculty, administrators, and food service personnel. Inclusion of all stakeholders in an organized communication plan developed in collaboration with the school and directed by an internal leader may result in increased awareness of the quality of the school nutrition environment and enhance support for healthy changes in the food venues. Food service personnel should be provided with opportunities to increase their knowledge about nutrition and health as well as training to implement simple changes in food preparation to provide healthier choices in schools.

Sustainability of the environmental change is more likely if a school nutrition policy is adopted. A written policy may help schools maintain healthy food choices during times of transition, such as turnover in school administrative or food service personnel and shifts in food preferences among faculty and students. A committee that includes stakeholders and selected decision makers may be best positioned to accomplish this.

Follow-up data will provide insight into the impact of the nutrition environmental change on student variables. The authors anticipate that the findings will provide additional support for adopting a school nutrition policy on competitive foods to sustain healthy changes.
